# Mpox Vaccination and Diagnosis Among Sexual and Gender Diverse Populations in Brazil: Results From a Large Nationwide Online Survey

**DOI:** 10.1002/jia2.70143

**Published:** 2026-07-24

**Authors:** Mayara Secco Torres Silva, Paula Mendes Luz, Carolina Coutinho, Brenda Hoagland, Emilia Moreira Jalil, Cristina Pimenta, Marcos Benedetti, Sandra Wagner Cardoso, Valdilea Gonçalves Veloso, Beatriz Grinsztejn, Thiago Silva Torres

**Affiliations:** ^1^ Instituto Nacional de Infectologia Evandro Chagas Fundação Oswaldo Cruz Rio de Janeiro Brazil

**Keywords:** Brazil, mpox, sexually transmitted infections, vaccination

## Abstract

**Introduction:**

Since the onset of the 2022 multinational mpox outbreak, Brazil has reported one of the highest case burdens globally, disproportionately affecting sexual and gender diverse populations. Mpox vaccination efforts in Brazil have been limited by delayed procurement, restricted eligibility criteria and structural access barriers. Understanding vaccination use, correlates of uptake and characteristics of individuals previously diagnosed with mpox is essential to guide equitable prevention strategies.

**Methods:**

We conducted an online cross‐sectional survey between August and December 2024 among individuals recruited through social media and dating apps. Eligible participants were aged ≥18 years, identified as a sexual and gender diverse person, and residing in Brazil. Primary outcomes were previous mpox vaccination and mpox diagnosis. Sociodemographic, behavioural, internalized LGBTQIAPN+phobia and HIV/sexually transmitted infections (STIs) variables were collected. Adjusted logistic regression models identified independent factors associated with both outcomes.

**Results:**

Of 9546 respondents, most were cisgender men (91.8%), aged >30 years (81.5%) and residing in capital cities (77.2%). Overall, mpox vaccination coverage was 4.9%; nearly half received only one dose, and one quarter were vaccinated outside Brazil. Previous mpox diagnosis was reported by 2.1% of respondents, with 76.5% seeking healthcare and 7.0% requiring hospitalization. Factors associated with previous mpox vaccination were self‐identification as White (aOR = 1.36; 95% CI: 1.10−1.69), more than five sex partners (aOR = 1.29; 95% CI: 1.03–1.62), living with HIV (aOR = 3.40; 95% CI: 2.59–4.47), current/prior HIV pre‐exposure prophylaxis (PrEP) use (aOR = 1.88; 95% CI: 1.41–2.53) and internalized LGBTQIAPN+phobia score (aOR = 0.80; 95% CI: 0.71−0.90). Factors associated with previous mpox diagnosis were living in capital cities (aOR = 1.88; 95% CI: 1.17–3.20), more than five sex partners (aOR = 1.93; 95% CI: 1.35−2.79), stimulant drug use (aOR = 1.61; 95% CI: 1.14–2.25), any STI diagnosis (aOR = 2.41; 95% CI: 1.74–3.33), living with HIV (aOR = 3.73; 95% CI: 2.31–6.25) and current/prior PrEP use (aOR = 2.35; 95% CI: 1.43−4.01).

**Conclusions:**

Mpox vaccination among sexual and gender diverse populations in Brazil remains critically low. Findings highlight structural barriers, including vaccine scarcity, delayed rollout and stigma, that hinder equitable vaccine access. Integrating mpox vaccination into combined HIV/STI prevention services, adopting differentiated care delivery models and partnering with community‐led organizations are essential to expanding reach, improving dose completion and reducing health inequities during ongoing and future outbreaks.

## 1 Introduction

Since the multinational mpox outbreak emerged in 2022, the Americas region has been significantly affected. Brazil has reported the second‐highest number of confirmed mpox diagnoses globally, following the United States, underscoring the country's central role in the regional epidemic [1]. After a sharp decline in notifications throughout 2023, similar to trends observed in Europe and North America, surveillance data from 2024 indicate a resurgence of cases in Brazil. Although at a lower magnitude than during the initial outbreak, evidence suggests ongoing and continued low‐level community transmission [2].

Mpox disproportionately affected cis men who have sex with men (MSM), but also impacted cis and trans women, as well as other gender‐diverse identities [3, 4]. High rates of HIV and other sexually transmitted infections (STIs) have been documented among people with mpox, reflecting overlapping sexual and social networks and underlying structural vulnerabilities. Barriers to timely diagnosis, such as limited clinical recognition outside highly affected populations, stigma, discrimination, and the social and economic consequences of prolonged home isolation, likely exacerbate inequities in mpox outcomes. At the same time, surveys conducted during the outbreak demonstrated that many of those most affected proactively adopted protective behaviours, including temporary reductions in sexual partners and other modifications in sexual practices, as a community‐driven strategy to mitigate mpox transmission [3, 5, 6, 7].

Effective mpox prevention relies on a combination of behavioural, biomedical and structural strategies. Integrated sexual health services, including HIV and STI testing, play a critical role in early detection and linkage to care. Condom use may reduce, but does not fully prevent, mpox transmission due to the potential for skin‐to‐skin transmission. Clear, community‐centred communication that avoids reinforcing stigma is essential to support engagement in prevention, testing and care [8]. Vaccination has become a cornerstone of mpox prevention. While first‐generation smallpox vaccines may provide partial cross‐protection based on observational evidence, the third‐generation non‐replicating Modified Vaccinia Ankara‐Bavarian Nordic (MVA‐BN; Jynneos) vaccine has demonstrated effectiveness for both post‐exposure and pre‐exposure prophylaxis (PrEP). Emerging real‐world studies indicate substantial protection against symptomatic infection, particularly after completion of the two‐dose regimen [9, 10].

In Brazil, delays in vaccine acquisition and limited global availability of MVA‐BN doses restricted access to mpox vaccination, reflecting broader Global North–Global South inequities in vaccine distribution. Although Brazil has a strong National Immunization Program within a universal public health system (Sistema Único de Saúde [SUS]), rollout remains limited. Vaccination began only in March 2023, after the initial decline in reported cases, and initially prioritized people living with HIV with advanced immunosuppression to prevent severe disease [11, 12]. Even after subsequent eligibility expansions, coverage remained disproportionately low relative to the size of populations considered at increased risk. By the end of 2024, only 32,573 doses had been administered nationwide, increasing to 53,267 doses by May 2025, with nearly half delivered in the Southeast region. In both periods, approximately 40% of administered doses corresponded to complete two‐dose schedules. These figures remain far below the estimated population potentially eligible for vaccination, including approximately 150,000 people on PrEP and nearly one million people with HIV in Brazil. Thus, even accounting for constrained global vaccine supply, vaccination coverage remained substantially insufficient to meet the potential public health need. Consistent with this interpretation, recent studies among MSM in Brazil documented low levels of mpox vaccine hesitancy, suggesting that barriers to vaccine availability and access, rather than unwillingness to be vaccinated, were the primary drivers of low coverage [13].

This study aimed to describe mpox vaccination patterns and investigate sociodemographic and behavioural factors associated with previous mpox vaccination and prior mpox diagnosis among sexual and gender diverse populations in Brazil. Understanding these factors is essential to inform equitable prevention strategies in Brazil, given previous epidemiologic, structural and programmatic considerations. Although vaccination and prior diagnosis reflect distinct processes, including differences in healthcare access, vaccine availability and exposure dynamics, both outcomes may provide important insights into engagement with health services and access to prevention technologies among these populations.

## 2 Methods

### 2.1 Study Design and Participants

This was a cross‐sectional online study conducted between August and December 2024 in Brazil to assess the health, sexual behaviour, and knowledge of HIV and prevention in the context of the ImPrEP Project [14]. For this analysis, we focused on information related to previous mpox vaccination and mpox diagnosis. Participants were recruited via advertisements on dating apps (Scruff and Grindr) and social media (Instagram and Facebook), which contained a brief study description and a link to the online survey. They could also share the link with others through apps for messages (e.g. WhatsApp) or e‐mail. Participants who clicked on the link were directed to a secure online platform programmed on Alchemer (https://www.alchemer.com), where they provided informed consent before proceeding with the survey. Participants were eligible if they clicked “yes” to the informed consent form. Inclusion criteria were: (1) aged 18 years or older; (2) identifying as a sexual and gender diverse person; and (3) living in Brazil. Participants were excluded if (1) they answered any attention question incorrectly. [15], (2) they had duplicated IP addresses. For this analysis, we included participants who completed the questionnaire until the question related to mpox vaccination. The online survey was administered in Portuguese and took approximately 20 min to complete. Participants were assured of the confidentiality and anonymity of their responses. Only one response per IP address was allowed.

### 2.2 Outcomes

We assessed two primary outcomes: (1) previous mpox vaccination (“Have you received any vaccine for mpox?”) and (2) previous mpox diagnosis (“Have you ever been diagnosed with mpox?”). Both outcomes were treated as dichotomous variables. We additionally described information related to mpox vaccination (e.g. number of doses received, country where vaccination occurred) and to mpox diagnosis (e.g. access to healthcare services, hospitalization and adherence to isolation guidance).

### 2.3 Measures

The following variables were included in the analysis. Demographic characteristics encompassed age (mean [SD], and categorized as 18–30 and >30 years), gender identity (cis man, trans man, cis woman, trans woman, *travesti* and non‐binary or queer), sexual orientation (gay, bisexual, heterosexual or other), race (Asian, Black, Indigenous, *Pardo*/Mixed or White), educational level (primary, secondary or superior), region (North, Northeast, Central‐West, South and Southeast) and living in a capital city (yes and no). Income considered household income per month, stratified into low (up to two minimum wages), middle (>2 to 6 minimum wages) and high (>6 minimum wages). One minimum wage was equivalent to 270 USD (1518 BRL) in Brazil in 2024. Sexual behaviour was assessed by the number of sex partners (≤5 or >5; following categorization used in other studies [14, 16, 17]) and condomless sex in the past 6 months (yes/no). HIV status was self‐reported as positive, negative or unknown. HIV prevention was assessed by self‐reported experience with HIV PrEP, categorized as never, current or past use. Stimulant drug use was defined as the use of cocaine, crack or party drugs (e.g. ecstasy/MDMA) in the past 6 months (yes or no). Participants were asked whether they had received a diagnosis of any STI (including syphilis, gonorrhoea, chlamydia, hepatitis B or others) from a healthcare provider in the past 6 months (yes or no).

The Reactions to Homosexuality Scale (RHS) was developed to assess negativity among MSM. The RHS has been validated in Portuguese among MSM from Brazil [18]. For this study, we adapted the scale to include all sexual and gender diverse populations, following a previous study [5, 19]. The adapted RHS demonstrated good internal consistency in this sample (alpha Cronbach = 0.82). The RHS consists of 7 items (example items include: “I feel comfortable discussing my sexuality in a public situation” and “Homosexuality is as natural as heterosexuality”) rated on a 7‑point Likert scale ranging from strongly disagree to strongly agree [18, 19]. Total scores range from 0 to 42; higher scores reflect greater levels of internalized LGBTQIA+ phobia.

### 2.4 Statistical Analysis

We described the total population and compared participants’ characteristics according to previous mpox vaccination and mpox diagnosis. Comparisons between groups were conducted using chi‐squared or Fisher's exact tests for categorical variables and Mann–Whitney test for discrete variables. We used logistic regression models to identify factors associated with both outcomes. Variables were included in the adjusted model regardless of significant *p*‐value thresholds in univariate analysis. Due to the limited sample size in some categories, we dichotomized the following variables to build the models. Gender was grouped as cis man versus transgender/non‐binary person, as the latter experience higher levels of stigma and discrimination in healthcare access [20, 21]. Race was grouped as White versus Asian/Black/Indigenous/*Pardo*, reflecting racial inequities in health outcomes in Brazil, where non‐White populations face greater structural barriers to prevention and care [22]. Region was grouped as North, Northeast and Central‐west versus Southeast and South, reflecting regional similarities. We created a dummy variable based on HIV status and PrEP use, categorizing into living with HIV, current or prior PrEP use and never used PrEP. Internalized LGBTQIAPN+phobia score was standardized to z‐scores for inclusion in regression models. As a sensitive analysis, we conducted a logistic regression model for mpox vaccination considering two or more doses. Results are presented as odds ratios (ORs) and adjusted odds ratios (aORs) with 95% confidence intervals (CIs). All analyses were performed using the R project version 4.5.2.

### 2.5 Ethical Considerations

The study was approved by the Instituto Nacional de Infectologia Evandro Chagas (INI)‐Fiocruz institutional review board (#CAAE 82021918.0.0000.5262) in Brazil. Informed consent was obtained from all participants prior to their participation in the survey. Participants received no compensation.

## 3 Results

From August to December 2024, a total of 22,406 persons accessed the questionnaire, 20,801 signed the electronic consent and 2726 were excluded (cisgender women who have sex only with men: 150; cisgender men who have sex only with women: 991; aged < 18 years: 40; living abroad: 28; incorrect attention question: 790; duplicated IP: 727). Of 18,075 eligible participants who initiated the questionnaire, 9546 completed the mpox vaccination question and were included in this analysis.

Overall, the mean age was 40.8 (SD 10.9) years (Table 1). Participants were predominantly cis men (91.8%), aged 30 or older years (81.5%), gay (77.5%) and self‐identified as White (55.6%). Most lived in the South/Southeast region (73.6%), had superior education (73.7%) and resided in capital cities (77.6%). Most reported five or less sex partners (61.1%) and condomless sex (68.4%) in the past 6 months. Overall, 24.5% reported a previous HIV diagnosis, and 35.4% reported current or prior PrEP use among persons reporting HIV negative/unknown status (*n* = 6974). Only 15.1% and 14.9% reported any stimulant drug use and any STI in the past 6 months, respectively.

**TABLE 1:** Characteristics of participants overall and according to self‐reported previous mpox diagnosis and vaccination.

	*N* = 9546	Previous mpox vaccination			Previous mpox diagnosis		
		No (%)	Yes (%)		No (%)	Yes (%)	
		9078 (94.8)	468 (4.9)	*p* value	9346 (97.9)	200 (2.1)	*p* value
**Age (years)**							
Mean (SD)	40.8 (10.9)	40.8 (10.9)	40.5 (10.2)	0.49	40.8 (10.9)	39.7 (8.0)	0.14
18−30	1762 (18.5)	1688 (18.6)	74 (15.8)	0.13	1736 (18.6)	26 (13.0)	0.044
**>** 30	7784 (81.5)	7390 (81.4)	394 (84.2)		7610 (81.4)	174 (87.0)	
**Gender**				0.007			0.19
Cis man	8759 (91.8)	8319 (91.6)	440 (94.0)		8566 (91.7)	193 (96.5)	
Trans man	56 (0.6)	55 (0.6)	1 (0.2)		56 (0.6)	0 (0)	
Cis woman	121 (1.3)	119 (1.3)	2 (0.4)		121 (1.3)	0 (0)	
Trans woman	54 (0.6)	51 (0.6)	3 (0.6)		54 (0.6)	0 (0)	
*Travesti*	21 (0.2)	17 (0.2)	4 (0.9)		21 (0.2)	0 (0)	
Non‐binary or queer	535 (5.6)	517 (5.7)	18 (3.8)		528 (5.6)	7 (3.5)	
**Sexual orientation**				0.004			<0.001
Gay	7401 (77.5)	7018 (77.3)	383 (81.8)		7224 (77.3)	177 (88.5)	
Bisexual	1381 (14.5)	1321 (14.6)	60 (12.8)		1362 (14.6)	19 (9.5)	
Heterosexual	449 (4.7)	442 (4.9)	7 (1.5)		449 (4.8)	0 (0)	
Other	315 (3.3)	297 (3.3)	18 (3.8)		311 (3.3)	4 (2.0)	
**Race**				0.099			0.007
Asian	109 (1.1)	105 (1.2)	4 (0.9)		105 (1.1)	4 (2.0)	
Black	1156 (12.1)	1099 (12.1)	57 (12.2)		1123 (12.0)	33 (16.5)	
Indigenous	72 (0.8)	68 (0.7)	4 (0.9)		67 (0.7)	5 (2.5)	
*Pardo* (mixed)	2901 (30.4)	2784 (30.7)	117 (25.0)		2846 (30.5)	55 (27.5)	
White	5308 (55.6)	5022 (55.3)	286 (61.1)		5205 (55.7)	103 (51.5)	
**Educational level**				0.94			0.025
Primary	282 (3.0)	268 (3.0)	14 (3.0)		274 (2.9)	8 (4.0)	
Secondary	2224 (23.3)	2118 (23.3)	106 (22.6)		2193 (23.5)	31 (15.5)	
Superior	7040 (73.7)	6692 (73.7)	348 (74.4)		6879 (73.6)	161 (80.5)	
**Income** ^a^				0.16			0.15
Low	2272 (23.8)	2156 (23.7)	116 (24.8)		2232 (23.9)	40 (20.0)	
Middle	4088 (42.8)	3907 (43.0)	181 (38.7)		4007 (42.9)	81 (40.5)	
High	3186 (33.4)	3015 (33.2)	171 (36.5)		3107 (33.2)	79 (39.5)	
**Brazilian Region**				0.019			0.019
North	286 (3.0)	271 (3.0)	15 (3.2)		285 (3.0)	1 (0.5)	
Northeast	1570 (16.4)	1509 (16.6)	61 (13.0)		1542 (16.5)	28 (14.0)	
Central‐west	664 (7.0)	616 (6.8)	48 (10.3)		641 (6.9)	23 (11.5)	
Southeast	6051 (63.4)	5759 (63.4)	292 (62.4)		5920 (63.3)	131 (65.5)	
South	975 (10.2)	923 (10.2)	52 (11.1)		958 (10.3)	17 (8.5)	
**Living in capital city**				0.057			< 0.001
No	2179 (22.8)	2089 (23.0)	90 (19.2)		2156 (23.1)	23 (11.5)	
Yes	7367 (77.2)	6989 (77.0)	378 (80.8)		7190 (76.9)	177 (88.5)	
**Sex partners** ^b^				< 0.001			< 0.001
≤ 5	5832 (61.1)	5594 (61.6)	238 (50.9)		5774 (61.8)	58 (29.0)	
> 5	3714 (38.9)	3484 (38.4)	230 (49.1)		3572 (38.2)	142 (71.0)	
**Condomless sex** ^b^				0.011			< 0.001
No	3018 (31.6)	2895 (31.9)	123 (26.3)		3000 (32.1)	18 (9.0)	
Yes	6528 (68.4)	6183 (68.1)	345 (73.7)		6346 (67.9)	182 (91.0)	
**Any stimulant drug use** ^b^ ^,^ ^c^				< 0.001			< 0.001
No	8103 (84.9)	7733 (85.2)	370 (79.1)		7971 (85.3)	132 (66.0)	
Yes	1443 (15.1)	1345 (14.8)	98 (20.9)		1375 (14.7)	68 (34.0)	
**Any STI diagnosis** ^b^ ^,^ ^d^ (*N* = 9416)				0.003			< 0.001
No	8011 (85.1)	7641 (85.3)	370 (80.3)		7896 (85.6)	115 (59.0)	
Yes	1405 (14.9)	1314 (14.7)	91 (19.7)		1325 (14.4)	80 (41.0)	
**HIV status** (*N* = 9423)				< 0.001			< 0.001
Negative	6470 (68.7)	6217 (69.4)	253 (54.5)		6374 (69.1)	96 (48.2)	
Positive	2309 (24.5)	2110 (23.6)	199 (42.9)		2209 (23.9)	100 (50.3)	
Unknown	644 (6.8)	632 (7.1)	12 (2.6)		641 (6.9)	3 (1.5)	
**PrEP use** (*N* = 6974)				< 0.001			< 0.001
Never	4503 (64.6)	4381 (65.2)	122 (47.1)		4476 (65.1)	27 (27.6)	
Current	1967 (28.2)	1849 (27.5)	118 (45.6)		1901 (27.6)	66 (67.3)	
Past	504 (7.2)	485 (7.2)	19 (7.3)		499 (7.3)	5 (5.1)	
Internalized LGBTQIAPN+phobia score^e^ Mean (SD)	10.9 (8.9)	11.0 (9.0)	8.8 (7.6)	< 0.001	10.9 (8.9)	8.7 (8.2)	< 0.001

Abbreviations: PrEP, pre‐exposure prophylaxis; STI, sexually transmitted infections.

^a^ Income considered household income per month, stratified into low (up to two minimum wages), middle (>2 to 6 minimum wages) and high (>6 minimum wages). One minimum wage was equivalent to 270 USD (1518 BRL) in Brazil in 2024.

^b^ Past 6 months.

^c^ Stimulant drugs were defined as the use of cocaine, crack or party drugs (e.g. ecstasy/MDMA).

^d^ Self‐reported diagnosis of any STI (including syphilis, gonorrhoea, chlamydia, hepatitis B or others) from a healthcare provider.

^e^ The Reactions to Homosexuality Scale adapted to measure internalized LGBTQIAPN+ phobia.

Overall, mpox vaccination was reported by 4.9% (Table 2). Nearly half of the participants who were vaccinated had received only one dose (46.4%), and 15.8% were vaccinated outside Brazil. A total of 200 (2.1%) participants reported a previous mpox diagnosis (Table 3). Among them, most reported following social isolation recommendation during mpox (89.0%), having no sexual activity during mpox (92.0%) and receiving healthcare support (76.5%). Mpox‐related hospitalization was low (7.0%), and the most reported reason was pain (78.6%).

**TABLE 2:** Mpox‐related vaccination characteristics among study participants.

	*n* (%)
Mpox vaccination (*n* = 9546)	
No	9078 (95.1)
Yes	468 (4.9)
Number of mpox vaccine doses (*n* = 468)	
One	217 (46.4)
Two	218 (46.6)
More than two	33 (7.1)
Year of mpox vaccination (*n* = 468)	
2022	89 (19.0)
2023	187 (40.0)
2024	85 (18.2)
Other	6 (1.3)
Unknown	101 (21.6)
Country of vaccination (*n* = 468)	
Brazil	394 (84.2)
USA	30 (6.4)
France	9 (1.9)
Canada	8 (1.7)
Other	27 (5.8)

**TABLE 3:** Mpox‐related diagnosis characteristics among study participants.

	*n* (%)
Mpox diagnosis (*n* = 9546)	
No	9346 (97.9)
Yes	200 (2.1)
Followed social isolation recommendations during mpox (*n* = 200)	
No	22 (11.0)
Yes	178 (89.0)
Reported sexual activity during mpox (*n* = 200)	
No	184 (92.0)
Yes	16 (8.0)
Received healthcare support during mpox (*n* = 200)	
No	47 (23.5)
Yes	153 (76.5)
Mpox‐related hospitalization (*n* = 200)	
No	186 (93.0)
Yes	14 (7.0)
Reasons for mpox‐related hospitalization (*n* = 14)	
Pain	11 (78.6)
Isolation	3 (2.3)
Skin infection treatment	6 (6.6)
Difficult to swallow	3 (2.3)
Complication on genital region	6 (6.6)

Compared with participants reporting no previous mpox vaccination, vaccination was more common among participants self‐identified as cis man (94.0% vs. 91.6%; *p* = 0.007), gay (81.8% vs. 77.3%; *p* = 0.004), reporting more than five sex partners (49.1% vs. 38.4%; *p*<0.001), stimulant drug use (20.9% vs. 14.8%; *p*<0.001), any STI diagnosis (19.7% vs. 14.7%; *p* = 0.003), living with HIV (42.9% vs. 23.6%; *p*<0.001) and currently using PrEP (45.6% vs. 27.5%; *p*<0.001). Internalized LGBTQIAPN+phobia score was lower among participants reporting previous mpox vaccination versus not (score: 8.8 [SD 7.6] vs. 11.0 [SD 9.0]; *p*<0.001).

Compared with participants with no mpox diagnoses, mpox diagnosis was more common among participants self‐identified as gay (88.5% vs. 77.3%; *p*<0.001), self‐identified as Black (16.5% vs. 12.0%; *p* = 0.007), with superior education (80.5% vs. 73.6%; *p* = 0.025), living in capital cities (88.5% vs. 76.9%; *p*<0.001), reporting more than five sex partners (71.0% vs. 38.2%; *p*<0.001), condomless sex (91.0% vs. 67.9%; *p*<0.001), any stimulant drug use (34.0% vs. 14.7%; *p*<0.001) and any STI diagnosis (41.0% vs. 14.4%; *p*<0.001) (Table 1). Mpox diagnosis was more frequent among people living with HIV (50.3% vs. 23.9%; *p*<0.001), and those currently using PrEP (67.3% vs. 27.6%; *p*<0.001). Internalized LGBTQIAPN+phobia score was lower among participants reporting previous mpox diagnosis versus not (score: 8.7 [SD 8.2] vs. 10.9 [SD 8.9]; *p*<0.001).

In adjusted regression model, factors associated with previous mpox vaccination were self‐identification as White (aOR = 1.36; 95% CI: 1.10−1.69), more than five sex partners (aOR = 1.29; 95% CI: 1.03–1.62), living with HIV (aOR = 3.40; 95% CI: 2.59–4.47), current/prior PrEP use (aOR = 1.88; 95% CI: 1.41–2.53) and internalized LGBTQIAPN+phobia score (aOR = 0.80; 95% CI: 0.71−0.90) (Table 4). In a sensitive analysis considering two or more doses of mpox vaccine, associations remained for living with HIV (aOR = 4.69; 95% CI: 3.18–7.03), current/prior PrEP use (aOR = 2.72; 95% CI: 1.80–4.18) and internalized LGBTQIAPN+phobia score (aOR = 0.83; 95% CI: 0.70−0.97).

**TABLE 4:** Logistic regression models to access factors associated with previous mpox diagnosis and previous mpox vaccination.

	Previous mpox vaccination				Previous mpox diagnosis			
	Unadjusted models		Adjusted model		Unadjusted models		Adjusted model	
	OR	*p*‐value	aOR	*p*‐value	OR	*p*‐value	aOR	*p*‐value
**Age (years)**								
18−30	Ref.		Ref.		Ref.		Ref.	
**>** 30	1.22 (0.95−1.58)	0.13	1.06 (0.80−1.42)	0.71	1.53 (1.03−2.37)	0.046	1.26 (0.81−2.05)	0.33
**Gender**								
Cis man	Ref.		Ref.		Ref.		Ref.	
Transgender/non‐binary person	0.70 (0.46−1.01)	0.070	0.78 (0.49−1.19)	0.28	0.40 (0.17−0.79)	0.017	0.51 (0.18−1.15)	0.15
**Race**								
White	1.27 (1.05−1.54)	0.014	1.36 (1.10−1.69)	0.0056	0.84 (0.64−1.12)	0.24	0.82 (0.60−1.13)	0.23
Asian, Black, Indigenous, *Pardo*	Ref.		Ref.		Ref.		Ref.	
**Educational level**								
Primary	Ref.		Ref.		Ref.		Ref.	
Secondary	0.96 (0.56−1.77)	0.88	0.87 (0.47−1.77)	0.67	0.48 (0.23−1.14)	0.071	0.43 (0.17−1.32)	0.10
Superior	1.00 (0.60−1.80)	0.99	0.83 (0.45−1.67)	0.57	0.80 (0.42−1.79)	0.55	0.60 (0.25−1.76)	0.29
**Income** ^a^								
Low	Ref.		Ref.		Ref.		Ref.	
Middle	0.86 (0.68−1.10)	0.22	0.71 (0.54−0.93)	0.013	1.13 (0.77−1.67)	0.54	1.01 (0.66−1.59)	0.96
High	1.05 (0.83−1.35)	0.67	0.83 (0.62−1.11)	0.20	1.45 (0.97−2.10)	0.074	1.02 (0.65−1.64)	0.93
**Region**								
North, Northeast and Central‐west	Ref.		Ref.		Ref.		Ref.	
Southeast and South	0.99 (0.81−1.23)	0.96	0.85 (0.67−1.07)	0.16	1.02 (0.75−1.42)	0.90	0.97 (0.68−1.39)	0.84
**Living in capital city**								
No	Ref.		Ref.		Ref.		Ref.	
Yes	1.26 (1.00−1.60)	0.058	1.07 (0.83−1.40)	0.59	2.31 (1.52−3.67)	< 0.001	1.88 (1.17−3.20)	0.013
**Sex partners** ^b^								
≤ 5	Ref.		Ref.		Ref.		Ref.	
> 5	1.55 (1.29−1.87)	< 0.001	1.29 (1.03−1.62)	0.026	3.96 (2.92−5.42)	< 0.001	1.93 (1.35−2.79)	<0.001
**Condomless sex** ^b^								
No	Ref.		Ref.		Ref.		Ref.	
Yes	1.31 (1.07−1.63)	0.011	0.82 (0.63−1.08)	0.16	4.78 (3.03−8.05)	< 0.001	1.69 (0.98−3.11)	0.073
**Any stimulant drug use** ^b^ ^,^ ^c^								
No	Ref.		Ref.		Ref.		Ref.	
Yes	1.52 (1.20−1.91)	< 0.001	1.10 (0.84−1.41)	0.49	2.99 (2.21−4.01)	< 0.001	1.61 (1.14−2.25)	0.0056
**Any sexually transmitted infection diagnosis** ^b^ ^,^ ^d^								
No	Ref.		Ref.		Ref.		Ref.	
Yes	1.43 (1.12−1.80)	0.003	1.05 (0.81−1.37)	0.69	4.15 (3.09−5.54)	< 0.001	2.41 (1.74−3.33)	< 0.001
**HIV care and prevention status**								
Living with HIV	3.39 (2.69−4.28)	< 0.001	3.40 (2.59−4.47)	< 0.001	7.50 (4.97−11.73)	< 0.001	3.73 (2.31−6.25)	<0.001
Current or prior PrEP^e^ use	2.11 (1.64−2.71)	< 0.001	1.88 (1.41−2.53)	< 0.001	4.90 (3.18−7.79)	< 0.001	2.35 (1.43−4.01)	0.0056
Never used PrEP	Ref.		Ref.		Ref.		Ref.	
Internalized LGBTQIAPN+phobia score^f^ Mean (SD)	0.76 (0.68−0.85)	< 0.001	0.80 (0.71−0.90)	< 0.001	0.75 (0.63−0.89)	< 0.001	0.92 (0.76−1.09)	0.34

Abbreviations: aOR, adjusted odds ratio; OR, odds ratio; PrEP, pre‐exposure prophylaxis.

^a^ Income considered household income per month, stratified into low (up to two minimum wages), middle (>2 to 6 minimum wages) and high (>6 minimum wages). One minimum wage was equivalent to 270 USD (1518 BRL) in Brazil in 2024.

^b^ Past 6 months.

^c^ Stimulant drugs were defined as the use of cocaine, crack or party drugs (e.g. ecstasy/MDMA).

^d^ Self‐reported diagnosis of any STI (including syphilis, gonorrhoea, chlamydia, hepatitis B or others) from a healthcare provider.

^e^ HIV pre‐exposure prophylaxis.

^f^ The Reactions to Homosexuality Scale adapted to measure internalized LGBTQIAPN+ phobia.

Factors associated with previous mpox diagnosis were living in capital cities (aOR = 1.88; 95% CI: 1.17–3.20), more than five sex partners (aOR = 1.93; 95% CI: 1.35−2.79), any stimulant drug use (aOR = 1.61; 95% CI: 1.14–2.25), any STI diagnosis (aOR = 2.41; 95% CI: 1.74–3.33), living with HIV (aOR = 3.73; 95% CI: 2.31–6.25) and current/prior PrEP use (aOR = 2.35; 95% CI: 1.43−4.01).

## 4 Discussion

We conducted a large cross‐sectional study among more than 9500 sexual and gender diverse persons in Brazil. We found low reports of mpox vaccination and a disproportionately high burden of prior mpox diagnosis among groups structurally vulnerable to HIV and other STIs. Consistent with national surveillance data, previous mpox diagnosis clustered among people living with HIV, individuals reporting multiple sex partners, stimulant use and any STI: patterns that reflect interconnected social, sexual and structural networks of risk. Prior engagement with HIV prevention and care services, particularly PrEP use and HIV care, was strongly associated with both vaccination and mpox diagnosis. This may indicate that sexual health and HIV programmes serve as critical, trusted points of access, given their history of providing affirming, stigma‐aware care to sexual and gender diverse populations and their greater clinical familiarity with mpox [23, 24].

In our study, previous mpox vaccination was low, and almost half of the participants completed vaccination with two doses. This likely reflects constrained vaccine availability rather than individual hesitancy, consistent with national reports documenting restricted access during the initial rollout [11, 12]. Settings with broader vaccine availability previously reported higher vaccination coverage and better two‐dose completion, suggesting that uptake improves when vaccines are accessible and effectively publicized to affected communities [25]. Together, these data reinforce the persistent gap between willingness to vaccinate and actual access to vaccination in Brazil and other countries from the Global South [26, 27]. Despite the recognized strength of the Brazilian National Immunization Program, mpox vaccination coverage among populations most affected by the mpox outbreak remained low during the study period [11, 12]. Importantly, Brazilian studies among sexual and gender diverse communities have shown low levels of mpox vaccine hesitancy, indicating that the low uptake observed was likely associated with barriers to vaccine availability and access, rather than unwillingness to be vaccinated [13]. While mpox diagnostic testing was broadly incorporated into the Brazilian national health system, vaccine availability remained limited, reflecting broader Global North–Global South inequities in access to emerging health technologies. Initial vaccination strategies prioritized people living with HIV and other key populations, but limited supply constrained broader implementation [11, 12]. This, coupled with the high engagement of sexual and gender diverse communities in HIV prevention and high willingness to adopt protective behavioural changes during the mpox outbreak, highlights existing strengths and a critical opportunity to expand mpox vaccination through accessible, community‐centred strategies [5, 13].

Persistent stigma, discrimination and LGBTQIAPN+‐phobia within healthcare settings may further inhibit vaccination access. Our findings suggest that mpox vaccination was shaped not only by individual willingness, but also by broader structural and programmatic factors influencing access to prevention technologies and healthcare services. Internalized LGBTQIAPN+‐phobia emerged as an independent barrier to vaccination, possibly reflecting avoidance of sexual health services and concerns about stigma associated with seeking mpox‐related prevention. Conversely, lower internalized LGBTQIAPN+‐phobia among individuals with prior mpox diagnosis may indicate greater engagement with healthcare and community networks. These findings reinforce that strengthening mpox prevention cannot be separated from broader efforts to reduce stigma and improve affirming, community‐centred sexual healthcare. Expanding equitable vaccine uptake will likely require integration with HIV and sexual health services, community‐led outreach, low‐threshold delivery models and healthcare environments that actively address discrimination and LGBTQIAPN+‐phobia.

The observed association between self‐identifying as White and prior mpox vaccination underscores persistent inequities in access to prevention technologies in Brazil. Despite universal health system principles, structural racism continues to shape healthcare engagement, resource distribution and perceived acceptability of services [28]. Sexual and gender diverse populations of White race may experience fewer discriminatory encounters, greater trust in institutions and improved navigation of health systems, facilitating earlier or more frequent access to mpox vaccination. Conversely, sexual and gender diverse populations of other races, who are disproportionately affected by discrimination, socioeconomic exclusion, racism in clinical settings and reduced access to culturally affirming care [28, 29], were significantly less likely to have been vaccinated, despite facing comparable or higher behavioural and structural vulnerabilities for mpox.

Notably, almost one in five participants with prior mpox did not seek healthcare, highlighting persistent barriers potentially related to stigma, discrimination in health settings, concerns about isolation or limited familiarity with mpox outside specialized services. Compared with a previous nationwide online survey conducted during the 2022 outbreak, the proportion reporting prior mpox was substantially lower in the present study (5.6% [324/6236] in 2022 vs. 2.1% [200/9546] in 2024), likely reflecting reduced community transmission after the epidemic peak, when no mpox vaccines were yet available in Brazil [5, 30]. Prior mpox diagnosis was associated with living in capital cities, having more than five sexual partners, stimulant drug use, previous STI diagnosis, living with HIV and current or prior PrEP use. These factors may reflect both greater exposure risk and increased engagement with healthcare, testing and surveillance services, potentially increasing the likelihood of diagnosis.

This study has limitations. Data were self‐reported and, therefore, subject to recall and social desirability bias, particularly regarding sexual practices, HIV status, mpox diagnosis and vaccination history. Different levels of access to healthcare and mpox knowledge could impact mpox diagnosis, leading to misclassification. The online recruitment strategy through dating apps and social media, and overrepresentation of persons living in metropolitan areas, with high educational attainment, and a mean age of 40.8 years limit generalizability to other populations who may experience lower vaccination access. The cross‐sectional design does not allow causal inference or determination of the temporal relationship between mpox diagnosis and vaccination. Moreover, the finding that one quarter of vaccinated participants were immunized outside Brazil, combined with the urban, highly educated profile of our sample, suggests that our estimates may overstate national coverage. Real‐world vaccination uptake among sexual and gender diverse populations, particularly those with lower socioeconomic status or limited healthcare access, is likely even lower. We did not instruct participants to consult official vaccination documentation, introducing the risk of misclassification. Although we included all evaluated variables in adjusted logistic regression models regardless of a significant *p*‐value in univariate analyses, residual confounding by unmeasured variables (e.g. access to healthcare services) cannot be ruled out. Nevertheless, the large sample size, multi‐regional geographic representation and focus on priority populations provide meaningful insights into real‐world mpox prevention dynamics in Brazil.

## 5 Conclusions

Mpox vaccination among sexual and gender diverse populations in Brazil remained low during the study period, despite the disproportionate burden of mpox observed in these communities. Our findings suggest that access to vaccination was shaped not only by individual factors, but also by broader structural and programmatic conditions. This includes constrained vaccine availability, which is ultimately related to unequal global distribution of prevention technologies and persistent stigma within healthcare settings. At the same time, prior engagement with HIV prevention and care services was strongly associated with both vaccination and prior mpox diagnosis, highlighting the central role of integrated sexual health services in mpox prevention and response. Expanding equitable access to mpox vaccination will likely require community‐centred and low‐threshold delivery strategies integrated with HIV and STI services, alongside broader efforts to reduce stigma and strengthen preparedness for emerging infectious disease outbreaks in Global South settings.

## Author Contributions

MSTS, PML, BH, CP, MB, VGV, BG and TST conceived the study. MSTS, PML, EMJ and TST developed the methodology. MSTS and TST assessed and verified the data and performed the formal analysis. MSTS wrote the first draft. PML and TST developed the software and supervised the current analysis and manuscript preparation. CC, EMJ and CP conducted the investigation and data collection. EMJ validated the data. SWC, VGV and BG contributed to visualization and provided supervision. MB was responsible for funding acquisition, project administration and resources. PML, CC, BH, EMJ, CP, MB, SWC, VGV and BG reviewed the manuscript for important intellectual content. All authors reviewed and approved the final version of the manuscript.

## Conflicts of Interest

The authors declare no conflicts of interest.

## Data Availability

The study's final de‐identified dataset and dictionary will be made available with publication of the manuscript upon reasonable request. A proposal should be submitted to the corresponding author's e‐mail, who will evaluate and approve the request. No additional documents will be made available.
